# Multiple Origins of Secretagogin Expressing Cortical GABAergic Neuron Precursors in the Early Human Fetal Telencephalon

**DOI:** 10.3389/fnana.2020.00061

**Published:** 2020-09-02

**Authors:** Ayman Alzu’bi, Gavin J. Clowry

**Affiliations:** ^1^Biosciences Institute, Newcastle University, Newcastle upon Tyne, United Kingdom; ^2^Department of Basic Medical Sciences, Faculty of Medicine, Yarmouk University, Irbid, Jordan

**Keywords:** annexin V, cerebral cortex, ganglionic eminences, inhibitory interneurons, matrix metalloprotease 2, preoptic area, secretagogin, septum

## Abstract

Secretagogin (SCGN) which acts as a calcium signaling sensor, has previously been shown to be expressed by a substantial population of cortical GABAergic neurons at mid-gestation in humans but not in mice. The present study traced SCGN expression in cortical GABAergic neurons in human fetal forebrain from earlier stages than previously studied. Multiple potential origins of SCGN-expressing neurons were identified in the caudal ganglionic eminence (CGE) lateral ganglionic eminence (LGE) septum and preoptic area; these cells largely co-expressed SP8 but not the medial ganglionic eminence marker LHX6. They followed various migration routes to reach their target regions in the neocortex, insular and olfactory cortex (OC) and olfactory bulbs. A robust increase in the number of SCGN-expressing GABAergic cortical neurons was observed in the midgestational period; 58% of DLX2+ neurons expressed SCGN in the cortical wall at 19 post-conceptional weeks (PCW), a higher proportion than expressed calretinin, a marker for GABAergic neurons of LGE/CGE origin. Furthermore, although most SCGN+ neurons co-expressed calretinin in the cortical plate (CP) and deeper layers, in the marginal zone (MZ) SCGN+ and calretinin+ cells formed separate populations. In the adult mouse, it has previously been shown that in the rostral migratory stream (RMS), SCGN, annexin V (ANXA5), and matrix metalloprotease 2 (MMP2) are co-expressed forming a functioning complex that exocytoses MMP2 in response to calcium. In the present study, ANXA5 showed widespread expression throughout the cortical wall, although MMP2 expression was very largely limited to the CP. We found co-expression of these proteins in some SCGN+ neurons in the subventricular zones (SVZ) suggesting a limited role for these cells in remodeling the extracellular matrix, perhaps during cell migration.

## Introduction

Secretagogin is an EF-hand calcium-binding protein similar in sequence and structure to calbindin (CalB) and calretinin (CalR) however these proteins are likely to have different functions (Schwaller, 2009). Secretagogin (SCGN) has a very high affinity for Ca^2+^ and acts as a calcium sensor (Rogstam et al., 2007; Khandewal et al., 2017) whereas CalB and CalR have moderate to high affinity and could act as both sensors and buffers (Schmidt, 2012; Schwaller, 2014). SCGN is known to have an important role in exocytosis in certain cells, for instance, pancreatic beta cells and neuroendocrine cells of the hypothalamus (Wagner et al., 2000; Romanov et al., 2015; Yang et al., 2016). Pancreatic beta cells are known to co-express isoforms of glutamate decarboxylase (GAD) the GABA synthesizing enzyme (Kim et al., 1993). In the hypothalamus, SCGN is co-expressed by a subset of GABAergic parvocellular neurons but not exclusively by this subset (Romanov et al., 2015). A ground-breaking study (Raju et al., 2018) demonstrated that expression of SCGN by cortical GABAergic neurons during development originating from the caudal and lateral ganglionic eminence (CGE and LGEs) at mid-gestation is a feature of human but not the mouse. Furthermore, SCGN expression is developmentally regulated and peaks before birth (Raju et al., 2018) with a very low density of interneuron-like SCGN+ cells detected in the neocortex of adult human post mortem brain by immunohistochemistry (Gartner et al., 2001; Tapia-González et al., 2020). However, a much higher density of both cell body and neuropil staining is observed in the human hippocampal and parahippocampal formations (Attems et al., 2007; Tapia-González et al., 2020).

It has been postulated that SCGN expression may contribute to increased dendritic complexity exhibited by interneurons of CGE origin in primates, and evidence to support this was provided by forcing expression of SCGN in developing mouse cortical interneurons and demonstrating increased dendritic complexity in such neurons compared to controls (Raju et al., 2018). SCGN interacts with the SNARE (soluble *N*-ethylmaleimide-sensitive fusion attachment protein receptor) protein SNAP25 in response to binding calcium (Rogstam et al., 2007). Exocytosis is the mechanism by which a new cell membrane is added to neurite growth cones (Tsaneva-Atanasova et al., 2009; Zylbersztejn and Galli, 2011) involving synapse related SNARE proteins (Kunwar et al., 2011) thus it seems entirely plausible that SCGN could play a role in regulating neurite outgrowth.

In addition to neurite outgrowth, cell migration is another developmental process that could be under differential control between species. The developing human forebrain appears to manifest more routes of migration for interneuron precursors from ventral to dorsal telencephalon than the rodent, perhaps reflecting the larger cell numbers and greater distances involved (Clowry et al., 2018; Molnár et al., 2019). It has been shown in the rostral migratory stream (RMS) a pathway guiding neuroblast migration from subventricular zone to olfactory bulb throughout life (Lois and Alvarez-Buylla, 1994) there is a scaffold of SCGN expressing neurons that exocytose matrix metalloprotease 2 (MMP2) *via* activation of SCGN and recruitment of Annexin V (ANXA5). Furthermore, restructuring of the extracellular matrix by MMP2 promotes neuroblast migration (Hanics et al., 2017). Recent research has shown that in the early postnatal forebrain, nearby to the RMS, lies a dorsal migratory stream that supplies GABAergic neurons to late-developing prefrontal cortical areas in both humans (up to 5 months postnatally; Sanai et al., 2011; Paredes et al., 2016) and ferret (up to 90 days postnatally; Ellis et al., 2019). A large proportion of these interneurons express SCGN (Raju et al., 2018; Ellis et al., 2019).

Our previous study demonstrated that SCGN expression is not confined to GABAergic neurons in the developing human forebrain but is also, for instance, a transient feature (7–10 post-conceptional weeks) of human thalamic projection neuron development (Alzu’bi et al., 2019). We also noticed that SCGN is expressed by various groups of cells in the ventral telencephalon at this stage. The present study explores further these previously unreported early expression patterns of SCGN, revealing multiple origins for SCGN+ GABAergic neurons and tracing multiple migratory pathways to the cerebral cortex and olfactory bulbs. Also, we explored the co-expression of SCGN with annexin V (ANXA5) or matrix metalloprotease (MMP2) to understand better the potential functional roles of SCGN.

## Materials and Methods

### Human Tissue

Human fetal tissue from terminated pregnancies was obtained from the joint MRC/Wellcome Trust-funded Human Developmental Biology Resource (HDBR^1^; Gerrelli et al., 2015). All tissue was collected with appropriate maternal consent and approval from the Newcastle and North Tyneside NHS Health Authority Joint Ethics Committee. Fetal samples ranging in age from 6.5 to 19 post-conceptional weeks (PCW) were used; 2 at 6.5 PCW, 2 at 7.5 PCW, 2 at 8 PCW, 2 at 10 PCW, 3 at 12 PCW and 2 at 19 PCW. Ages were estimated from images of Carnegie stages provided by HDBR (up to 8 PCW) and foot and heel to knee length measurements according to Hern (1984) for later developmental stages. For immunostaining, brains were isolated and fixed for at least 24 h at 4°C in 4% paraformaldehyde (Sigma–Aldrich) dissolved in 0.1 M phosphate-buffered saline (PBS). Once fixed, whole or half brains (divided sagittally) were dehydrated in a series of graded ethanols before embedding in paraffin. Brain samples were cut at 8-μm section thickness in three different planes; horizontal, sagittal, and coronal, and mounted on slides.

### Immunohistochemistry

This was carried out on paraffin sections according to previously described protocols (Alzu’bi et al., 2017). Antigen retrieval involved boiling in 10 mM citrate buffer pH6 for 10 min. Sections were incubated with primary antibody [diluted in10% normal blocking serum in Tris-buffered saline (TBS) pH 7.6] overnight at 4°C. Details of primary antibodies are found in Table 1. The sections were incubated with biotinylated secondary antibody for 30 min at room temperature (Vector Laboratories Limited, Peterborough, UK) 1:500 dilution in 10% normal serum in TBS followed by incubation with avidin-peroxidase for 30 min (ABC-HRP, Vector Labs) then developed with diaminobenzidine (DAB) solution (Vector Labs), washed, dehydrated, and mounted using DPX (Sigma–Aldrich, Poole, UK). For double immunofluorescence, the Tyramide Signal Amplification (TSA) method was used, permitting double-staining using same-species antibodies. At the secondary antibody stage, sections were incubated with HRP-conjugated secondary antibody for 30 min [ImmPRESS HRP IgG (Peroxidase) Polymer Detection Kit, Vector Labs] and then incubated in the dark for 10 min with fluorescein tyramide diluted at 1/500 TSA fluorescein plus system reagent (Perkin Elmer, Buckingham, UK) leaving fluorescent tags covalently bound to the section. The sections were then boiled in 10 mM citrate buffer pH 6 to remove all antibodies and unbound fluorescein incubated first in 10% normal serum and then with the second primary antibody for 2 h at room temperature. The sections were again incubated with an HRP-conjugated secondary antibody followed by CY3 tyramide for 10 min (TSA CY3 plus system reagent, Perkin Elmer). The sections were counterstained with 4,6-diamidino-2-phenylindole dihydrochloride (DAPI; Thermo Fisher Scientific, Cramlington, UK) and mounted using Vectashield Hardset Mounting Medium (Vector Labs). Extensive washing of sections was carried out between all incubations.

**Table 1:** Primary antibodies used in this study.

Primary antibody	Antigen	Species	Dilution	Supplier	RRID number (where available)
**SCGN** secretagogin	Recombinant protein fragment. See https://www.proteinatlas.org/ENSG00000079689-SCGN/antibody	Rabbit Pycl	1/500	Sigma–Aldrich, Poole, UK	AB_1079874
**ANXA5** Annexin V	Purified human Annexin V.	Mouse Mncl	1/200	Sigma–Aldrich	AB_476772
**MMP2** Matrix metalloprotease	Chinese hamster ovary cell line CHO-derived recombinant human MMP-2	Mouse Mncl	1/100	R&D Systems, Abingdon, UK.	AB_358834
**GAD67** Glutamate decarboxylase 67 kD	Recombinant GAD67 protein Human, rat, mouse-specific	Mouse Mncl	1/1,000	Merck Millipore, Watford, UK	AB_2278725
**DLX2** Distal-less homeobox 2	Amino acids 211–250 mapping within the internal region of human DLX2.	Mouse Mncl	1/200	Santa Cruz, Heidelberg, Germany	Catalog number SC393879
**SP8** Specificity protein 8	Human SP8	Goat Pycl	1/500	Santa Cruz	AB_2194626
**Calretinin**	Recombinant rat calretinin	Mouse Mncl	1/500	Merk Millipore	AB_94259
**COUP-TFII** Chicken ovalbumin upstream promoter transcription factor 2	Recombinant human COUP-TF II/NR2F2 Amino Acids 43–64	Mouse Mncl	1/500	R&D Systems, Abingdon, UK	AB_2155627
**NKX2.1** NK2 homeobox 1	Recombinant rat TTF-1 (NKX2.1)	Mouse Mncl	1/150	Dako, Ely, UK	Catalog number M3575
**LHX6** LIM/homeobox protein 6	Amino acids 1–75 mapping at the N-terminus of LHX6 of human origin	Mouse Mncl	1/200	Santa Cruz	AB_10649856

### RNAseq

Full details of the origins, collection, preparation, sequencing, and analysis of the human fetal RNA samples have been previously described (Lindsay et al., 2016; Harkin et al., 2017). Temporal lobes were removed and, in larger brains, divided into frontal and posterior sections; the remaining cortex was cut into coronal slices usually 5 mm wide containing both medial and lateral wall. The entire RNAseq dataset from which data were extracted for this study has been deposited at www.ebi.ac.uk/arrayexpress/experiments/E-MTAB-4840. High-quality reads were then mapped to the human reference genome hg38 with Tophat2 (Kim et al., 2013). Reads aligned to genes and exons were counted with htseq-count (Anders et al., 2014) and normalized RPKM calculated. Read length was 101 bp before trimming and 85 bp after trimming with no reads of <20 bp retained. The minimum number of reads examined per sample was 63 million (average 90 million).

### Imaging and Cell Quantification

Images from immunoperoxidase-stained sections were captured using a Leica slide scanner and Zeiss Axioplan 2 microscope, and from immunofluorescent-stained sections with a Zeiss Axioimager Z2 apotome. Images were adjusted for brightness and sharpness using Adobe PHOTOSHOP CS6 software. Double labeling of cells for SCGN with either DLX2 or CalR was counted from five sections selected at intervals along the anterior-posterior axis of sections from two samples aged 19 PCW. The sections were observed under medium magnification; rectangular counting boxes 100 μm in width were placed over the ventricular/subventricular zones (VZ/SVZ) and intermediate zone/cortical plate (IZ/CP) delineated by the nuclear staining (DAPI).

## Results

### SCGN Expression in the Forebrain 6.5 PCW–8 PCW

**Figure 1:**
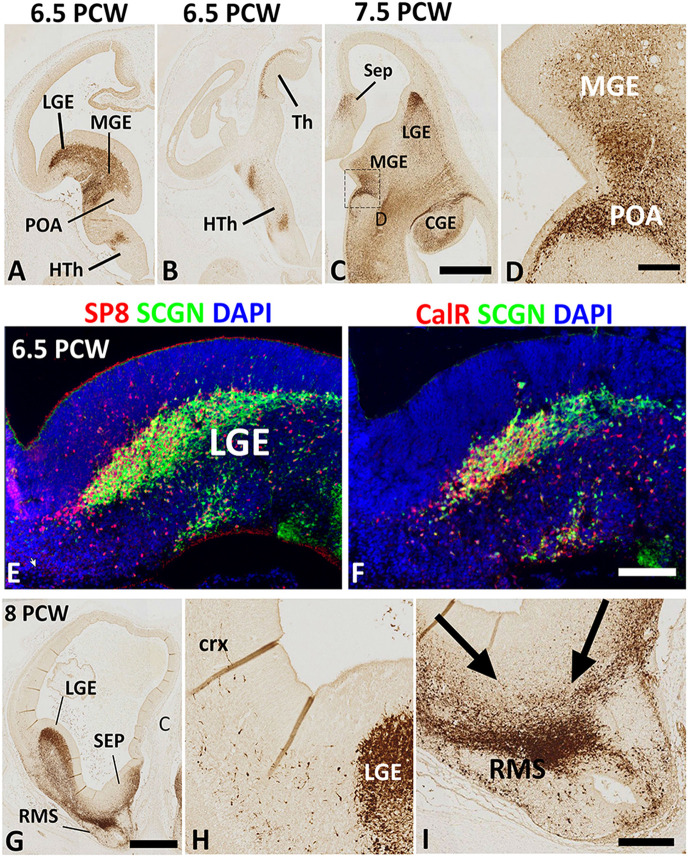
Secretagogin (SCGN) expression 6.5–8 post-conceptional weeks (PCW). **(A)** Rostral coronal section of 6.5 PCW brain; intense SCGN expression in ganglionic eminences (GE) preoptic area (POA) basal telencephalon and hypothalamus (HTh) and **(B)** Caudal thalamus (Th) and several locations in the hypothalamus. **(C)** Horizontal section of 7.5 PCW brain; SCGN immunoreactivity in the medial ganglionic eminence (MGE), lateral ganglionic eminence (LGE), caudal ganglionic eminence (CGE), septum (SEP), and POA. **(D)** A high magnification view of the boxed area in **(C)** shows many SCGN+ cells that appeared to enter MGE from POA. **(E)** Double labeling for SCGN and specificity protein 8 (SP8) in LGE showing most SCGN+ cells were double-labeled with SP8, although there is a population of SP8+ only cells in the dorsal most LGE. **(F)** Double labeling for SCGN and calretinin (CalR) in LGE showing only a proportion of SCGN+ cells co-expressed CalR. **(G–I)** The rostral coronal section at 8 PCW showing intense SCGN expression in LGE, septum, and rostral migratory stream (RMS, **G**). Few SCGN+ cells enter cortex from LGE **(H)**. Cells from LGE and septum appeared to be mainly migrating into RMS at this stage **(I)**. Scale bars: 1 mm in **(C)** (and for **A**,**B**) and **(G)**, 50 μm in **(D,F)** (and for **E**), and **(I)** (and for **H**).

The earliest stage studied here was 6.5 PCW when the prelate initially emerges (Meyer et al., 2000). Intense SCGN expression was mainly restricted to the ventral telencephalon including the post-mitotic zones of the LGE, medial ganglionic eminence (MGE), and anterior entopeduncular nucleus/preoptic area (AEP/POA) with no expression seen in the dorsal telencephalon (Figure 1A). Besides, this stage was also marked the initial emergence of early-born SCGN+ post-mitotic neurons of the outer mantle layer of the dorsal thalamus (Figure 1B; Alzu’bi et al., 2019). Although SCGN+ cells were also observed in the post-mitotic zones of the MGE (Figures 1A,C), double immunofluorescence for SCGN with pre- (NKX2.1) and post-mitotic (LHX6) markers of MGE-derived cells (Butt et al., 2008; Vogt et al., 2014; Sandberg et al., 2016) showed they were expressed in separate populations of cells (data not shown) indicating that SCGN+ cells are not likely to be generated in the MGE, instead, they could have been born in the region of the telencephalic stalk (POA; Figure 1D), and have migrated dorsally *via* MGE toward the LGE and dorsal telencephalon. SCGN+ cells were also observed in the post-mitotic layer of the sub-pallial septum (Figure 1C) predominantly in the LGE-like as opposed to MGE-like compartment (Alzu’bi et al., 2017). To explore the identity of SCGN+ cells in the various domains of the ventral telencephalon, double immunofluorescent labeling demonstrated that most SCGN+ cells co-expressed the transcription factor SP8 (Figure 1E) and a proportion co-expressed CalR (Figure 1F). Both SP8 and CalR are markers of human CGE/dLGE derived GABAergic neurons (Reinchisi et al., 2012; Hansen et al., 2013; Ma et al., 2013; Alzu’bi et al., 2017).

Eight PCW marked the onset of SCGN immunoreactivity in the human fetal cerebral cortex. At this stage, a small number of SCGN+ cells appeared to be entering the cortex from either the LGE/CGE (into the lateral and dorsal cortical wall) or the septum (into the medial cortical wall; Figures 1G,H). On the other hand, a much larger number of cells from ganglionic eminences (GE) and septum appeared to be migrating ventro-rostrally into the (RMS; Figure 1I) migrating towards the olfactory bulb. Migratory behavior in groups of cells was assumed if pronounced leading and trailing processes were orientated in the same direction and there was the decreasing density of such cells from the proposed origin to the proposed target of the migrating cells.

### SCGN Expression in the Forebrain 10–12 PCW

**Figure 2:**
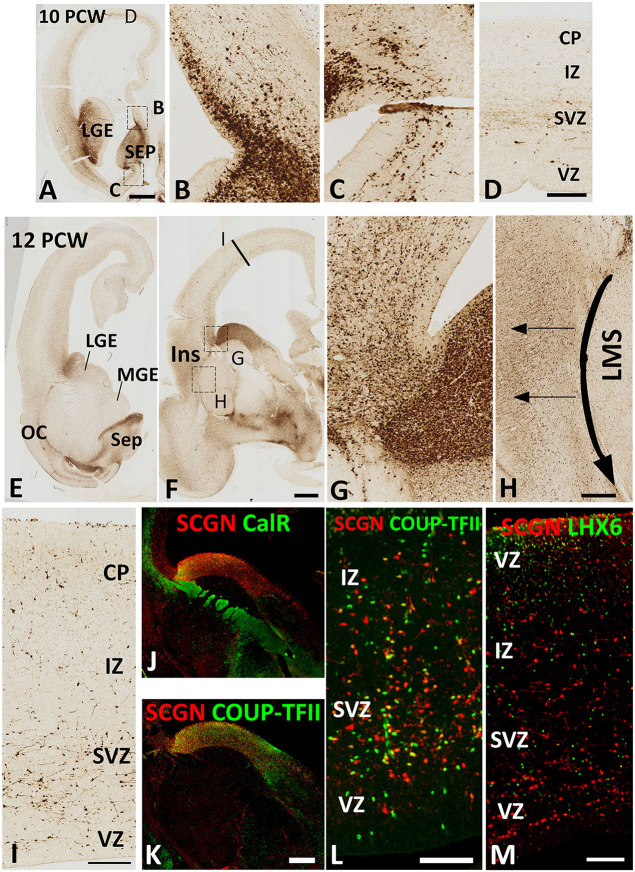
SCGN expression 10–12 PCW. At 10 PCW **(A)** high expression observed in LGE and SEP. Higher magnification insets show the migration of SCGN+ cells from septum into dorsomedial **(B)** and ventromedial **(C)** cortex. In dorsal cortex **(D)** a small number of SCGN+ cells seen predominantly migrating through subventricular zone (SVZ). By 12 PCW **(E,F)**, SCGN+ expression concentrated in dorsal LGE and low in MGE with higher magnification inset showing SCGN+ cells crossing the pallial/subpallial border to invade lateral cortex **(G)** and also migrating ventrolaterally in the lateral migratory stream (LMS) towards olfactory cortex (OC) and amygdala **(E,F**). Some SCGN+ exit LMS to populate insula (Ins, **F**,**H**). In the dorsal cortex **(I)** there are increased numbers of SCGN+ cells compared to 10 PCW and throughout the cortical wall while still favoring the SVZ. A proportion of SCGN+ cells in the LGE co-express CalR **(J)** but a higher number still co-express COUP-TFII both in the LGE **(K)** and the cortical wall **(L)**. However, no co-expression with LHX6 observed **(M)**. Scale bars: 1 mm in **(A)** (and for **E**); 50 μm in **(D)** (and for **B,C**); in **(H,I,L,M)**, and in **(K)** (and for **J**).

By 10 PCW, as an expression of SCGN is downregulated in the thalamocortical afferents (Alzu’bi et al., 2019) many SCGN+ cells had migrated into the cerebral cortex from the GE (Figure 2A). SCGN+ cells from the septum were also observed entering the dorsal and ventral medial wall of the anterior cortical regions (Figures 2B,C). However, SCGN+ cells in the cortical wall were mainly restricted to migratory cells in the SVZ; few were seen in the CP at this stage (Figure 2D).

By 12 PCW, an extremely dense population of SCGN+ cells entered the cortex from LGE/CGE (Figures 2E–G). SCGN+ cells also appeared to be following the curvature of the pallial/subpallial boundary crossing the internal capsule in the lateral migratory stream (LMS; Figure 2H) possibly guided by the radial glia fiber fascicle that is present at the boundary before they change direction to migrate radially into the future olfactory and insular cortical areas (González-Arnay et al., 2017). The CP, even in dorsal cortical regions, became more populated with SCGN+ cells. Also, the VZ/SVZ provided a potential migratory route for many SCGN+ neurons at this stage of development (Figure 2I).

Double-labeling experiments for SCGN with either CalR or COUP-TFII, a marker for CGE derived GABAergic neurons (Alzu’bi et al., 2017) showed the only proportion of SCGN+ cells in the LGE/CGE co-expressed CalR (Figure 2J), whereas the majority co-expressed COUP-TFII. Similar findings were also observed in the cortical wall (Figures 2K,L). No SCGN/LHX6 double labeling was observed in any region of the cortical wall (Figure 2M) confirming that the MGE is not a source of SCGN expressing cortical GABAergic interneurons.

### Robust Increase in SCGN Expressing GABAergic Neurons During the Midgestational Period

**Figure 3:**
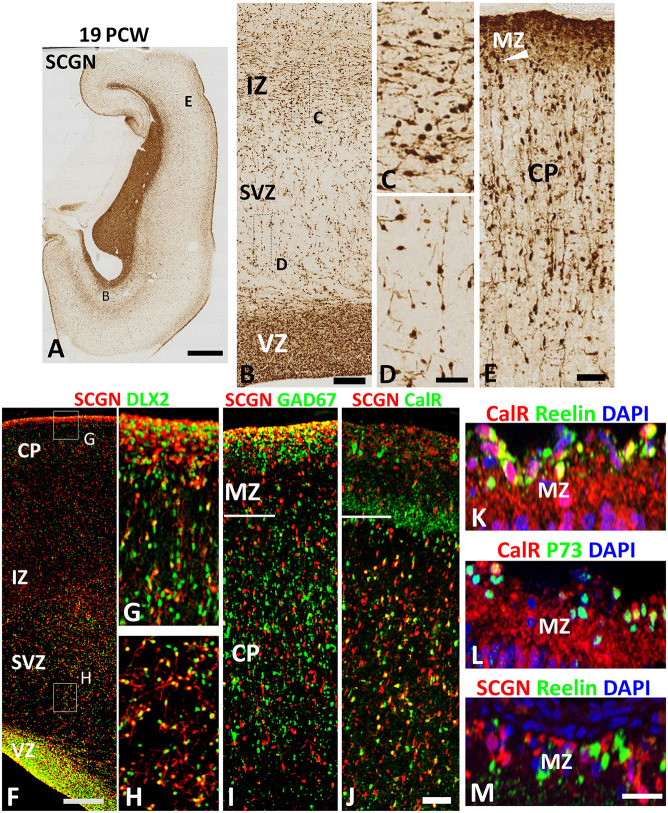
SCGN expression at 19 PCW. **(A)** The coronal section at 19 PCW with intense SCGN expression in CGE but also in ventricular zone (VZ) of the cortical wall **(B)** and in the marginal zone (MZ, **E**) otherwise moderate expression throughout the cortical wall. Panels **(B–D)** show that SCGN+ cells exhibit morphology of predominantly tangentially migrating cells in intermediate zone (IZ) but are sparser in SVZ, unlike 10–12 PCW, and show predominantly radial migratory morphology. In the cortical plate (CP) SCGN+ cells also have predominantly radial migratory morphology **(E)**. **(F)** A high proportion of SCGN+ neurons co-expressed DLX2 in both CP/MZ **(G)** and SVZ **(H)**. SCGN+ neurons in MZ, in particular, strongly co-expressed GAD67 **(I)**. Co-expression with CalR was abundant in the CP but in MZ, CalR and SCGN showed largely separate patterns of expression **(J)**. In the outer, subgranular layer of the MZ, CalR primarily co-localized with markers of Cajal Retzius cells, Reelin and P73 **(K,L)** whereas SCGN did not **(M)**. Scale bars: 2 mm in **(A)**; 100 μm in **(B)**; 50 μm in **(D)** (and for **C**) **(F,J)** (and for **G–I**) and in **(M)** (and for **L,K**).

SCGN expression was also evaluated in the cortical wall of the human fetal brain around the mid-gestational period. A robust increase in the number of SCGN+ cells was observed in the cortical VZ at 19 PCW (Figures 3A,B) as has previously been described for DLX2+ and CalR+ neurons at this developmental stage (Alzu’bi and Clowry, 2019). SCGN+ cells in the SVZ mainly showed a migratory morphology that suggested they were most likely following a radial path from the VZ towards the CP; whereas a stream of cells at the interface between the IZ and SVZ were mainly horizontally oriented perhaps representing tangentially migrating cells (Figures 3B–D). In the CP, SCGN+ cells were radially oriented and exhibited a bipolar morphology, with a large number of SCGN+ cells also appearing to descend from the MZ into the CP (Figure 3E) indicating the MZ could be also considered as a migration route for SCGN expressing cortical GABAergic neurons. Including the MZ, a majority of SCGN+ cells in the cortical wall co-expressed DLX2 (Figures 3F–H) and many co-expressed GAD67 (Figure 3I) confirming that SCGN is predominantly expressed in GABAergic neurons in the human cerebral cortex, as previously shown (Raju et al., 2018).

To further differentiate the populations of SCGN+ cells from CalR+ cells we found that proportion of SCGN+ cells in the CP, but not the MZ, co-expressed CalR (Figure 3J) confirming a distinct cellular identity for SCGN+ interneurons in the MZ. CalR+ cells in the MZ include Cajal-Retzius cells (Meyer et al., 2000) that co-express their markers Reelin and P73 (Figures 3K,L) whereas SCGN+ cells were exclusively GABAergic neurons co-expressing GAD67 but not Reelin (Figures 3I,M). Reelin is also a marker of some cortical inhibitory interneurons, including neurogliaform (NGCs) and single bouquet-like cells (SBCs) in layer I of the adult rodent neocortex (Jiang et al., 2015; Cadwell et al., 2016) indicating these cell types may not express SCGN. Finally, to investigate the proportions of cortical GABAergic neurons that express CalR and SCGN in the cortical wall, we counted the number of DLX2+ cells that co-expressed each protein. A higher proportion of DLX2+ GABAergic neuron precursors expressed SCGN than CalR; 58% (±3%; standard error of the mean) of interneuron precursors co-expressed SCGN, compared to 45% (±2%) expressing CalR.

### The Co-expression of Secretagogin With Either Annexin V or Matrix Metalloprotease-2

**Figure 4:**
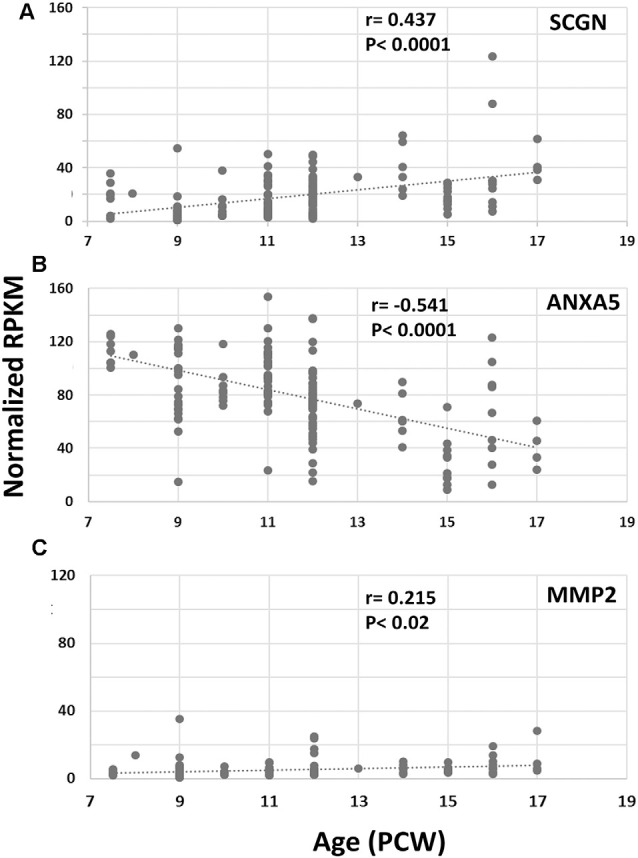
RNAseq for cortical expression of *SCGN, ANXA5* and *MMP2*, 7.5–17 PCW. Changes of expression (normalized RPKM) with age. *SCGN* expression showed steady and statistically significant increases with age **(A)** whereas *ANXA5* showed a significant decrease in expression **(B)**. *MMP2* was modestly expressed at all ages studied but expression increased to a small but significant degree with age **(C)**. Correlation coefficients (*r*) and *p*-values for *r* (*p*) are displayed on the charts.

Finally, we set out to explore one potential role of SCGN in the transduction of calcium signaling *via* ANXA5 to evoke the release of MMP2. Using RNAseq data (Lindsay et al., 2016,^2^) from samples from 7.5 to 17 PCW human fetal brain and immunohistochemical analysis, we first identified the expression level of both ANXA5 and MMP2 and explored their presence in SCGN+ cells. *SCGN* mRNA expression levels significantly increased with age (Figure 4A) in agreement with our immunohistochemical data (Figures 1–3). Expression appeared higher in the frontal and temporal cortex at all ages studied (not shown) in agreement with observations for other genes expressed by immature cortical GABAergic neurons (Molnár et al., 2019). The expression of *ANXA5* was generally higher than *SCGN* but decreased significantly with age (Figure 4B) and statistical tests for correlated expression of *SCGN* and *ANXA5* in tissue samples found no evidence for this. *MMP2* showed relatively low levels of expression with a slight but statistically significant trend towards increased expression with age (Figure 4C). Therefore, these observations found no evidence for widespread, correlated expression of these genes at the mRNA level in the developing human cerebral cortex.

**Figure 5:**
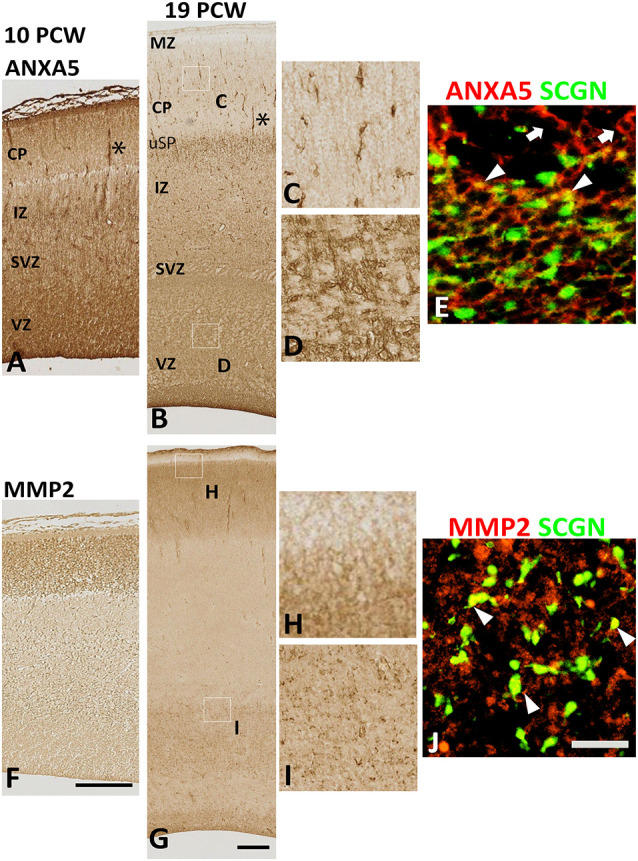
Expression of ANXA5 and MMP2 in the cortical wall. **(A)** At 10 PCW there was strong immunoreactivity for ANXA5 throughout cortical wall especially in MZ, VZ, and blood vessels (*). **(B)** MMP2 expression was entirely confined to CP. **(C)** By 19 PCW, ANXA5 expression was still strong in VZ, SVZ **(E)**, and blood vessels (*) but much reduced in MZ. However, many positive cells present in the upper subplate (uSP). Occasional cells with neuronal morphology in CP were also strongly immunostained for ANXA5 **(D)**. In SVZ, both SCGN+ (arrowheads) and SCGN− (arrows) cells expressed ANXA5 **(F)**. MMP2 expression was largely confined to CP (but not MZ, see **G,H**) but there was also expressed in the VZ and SVZ **(G,I)** where MMP2 immunoreactivity was found in close apposition to SCGN+ cells (arrowhead, **J**). MZ, marginal zone; CP, cortical plate; pSP, presubplate; uSP, upper subplate; lSP, lower subplate; IZ, intermediate zone; SVZ, subventricular zone; VZ, ventricular zone. Scale bars: 100 μm in **(F)** (and for **A**) in **(G)** (and for **B**); 50 μm in **(J)** (and for **C–E**, **H** and **I**).

Therefore, we turned to immunohistochemical analysis to see if the subsets of cells co-expressed these genes. There was an intense and widespread expression of ANXA 5 throughout the cortical wall at 10 PCW, although expression was strongest in the MZ, VZ, and in association with blood vessels (Figure 5A). Expression of MMP2, however, was restricted to the CP (Figure 5F). By 19 PCW, expression of ANXA5 appeared to have decreased slightly, in agreement with the RNAseq data. It was still strongly associated with blood vessels and also showed strong expression in a small population of cells in the CP and somewhat larger populations of cells in the proliferative zones (Figures 5B–D). Many ANXA5+ cells were present in the upper subplate, however, ANXA5 immunoreactivity was not observed in the MZ at this developmental stage (Figure 5B). By 19 PCW, the strongest expression of MMP2 was observed in the CP and pial surface but not in the MZ, with moderate expression also seen in the outer subventricular zone, and ventricular zone (VZ; Figures 5G–I).

At 19 PCW several SCGN+ cells co-expressed ANXA5 (Figure 5E), with also a proportion of SCGN+ cells expressing MMP2 on their extracellular surface (Figure 5J) however these two types of double-labeled cells were only observed in the VZ/SVZ which becomes a major tangential migratory route for GABAergic neuron precursors (post-mitotic, migratory) at this stage of development (Alzu’bi and Clowry, 2019) suggesting that SCGN could also have a role in GABAergic neuron migration in the human cortex. However, it is worth mentioning that neither the expression of ANXA5 or MMP2 was specific to SCGN+ cells but that they were also expressed by other cell types including dorsal progenitor cells and post-mitotic neurons of the CP which indicates the widespread role of MMP2 activity, possibly in neural migration in the developing human cortex, but not limited to SCGN+ cells.

## Discussion

In this study, we have confirmed that a substantial proportion of cortical GABAergic neurons express SCGN by mid-gestation in the developing human forebrain and shown that a higher proportion of cortical GABAergic neurons express SCGN than express CalR. Reporting on expression at younger stages for the first time, we observed that SCGN expression by GABAergic neuron precursors occurs at all stages of development, and we observed four major origins for SCGN+ GABAergic neurons, the CGE, LGE, subpallial septum, and preoptic area. SCGN+ cells predominantly co-expressed SP8 but not markers for cells of MGE origin. The earliest born SCGN+ cells appeared to mainly target the basolateral complex (*via* LMS) and the olfactory bulb (*via* RMS). After 8 PCW, large streams of SCGN+ cells also started migrating into the neocortex.

### Multiple Origins of SCGN Expressing Interneurons

These results suggest that the origin of SCGN expressing GABAergic neurons in the fetal human neocortex is not restricted to the CGE/LGE; the subpallial septum and POA also contribute to a proportion of these populations. We have recently discovered that the septum is a major contributor of GABAergic neuron precursors to the human cortex *via* medial migration routes not observed in rodent models (Alzu’bi et al., 2017; Alzu’bi and Clowry, 2019). The subpallial septum can be divided into MGE-like and LGE-like domains, based on gene expression patterns (Alzu’bi et al., 2017; Clowry et al., 2018) and as would be predicted, SCGN+ interneuron precursors arose from the more dorsal LGE-like domain.

In rodents, the VZ of the POA is the birthplace for about 10% of the total number of cortical interneurons. Although the comparatively small region of neurogenesis, it contributes a diversity of GABAergic neuron subtypes to the cerebral cortex (Gelman et al., 2009). They arise from two distinct sub-domains, the *Nkx2.5* expressing, more dorsal pPOA1 which gives rise to principally neurogliaform cells (Gelman et al., 2011; Niquille et al., 2018) and the *Dbx1* expressing, more ventral pPOA2 which gives rise to a great diversity of cell types including parvalbumin and somatostatin expressing neurons typical of MGE-derived interneurons (although they do not express *Lhx6*) and Reelin, Neuropeptide Y and some CalR expressing neurons typical of CGE-derived neurons (Gelman et al., 2011; Asgarian et al., 2019). In the present study, SCGN was expressed by neurons present in the mantle zone over both parts of the POA and could thus potentially be expressed by a wide range of interneurons during development. However, we found no evidence of the co-expression of neurogliaform cell markers in SCGN+ neurons in the MZ (Figure 3).

Primates display complexity in layer I of the cortex not found in rodents. In humans, around 11 PCW, the subpial granular layer (SGL) starts forming from cells observed to spread from the olfactory region to the nearby anterior/ventral cortex (Zecevic and Rakic, 2001; Meyer and González-Gómez, 2018). By 16 PCW the SGL covers the entire cortical surface of the forebrain, forming the most superficial part of the MZ (Meyer and González-Hernández, 1993) and consisting of small GABAergic cells previously reported to almost entirely co-express CalR (Meyer and González-Gómez, 2018) as well as large Reelin-positive Cajal–Retzius cells (Meyer et al., 2002). Small non-GABAergic CalR+ neurons are found in the deep MZ at the boundary with the CP (Meyer et al., 2000). We have found that there is a distinct population of SCGN+/GAD67+/CALR−neurons separate from the CALR+/GAD67+ neurons previously described. Some of the GABAergic cells of the SGL can undergo inward migration into the CP (Zecevic and Rakic, 2001; Rakic and Zecevic, 2003). Perhaps SCGN expression sorts inward migrating GABAergic neurons from the neurogliaform cells and small CR+ neurons that remain in the MZ.

By mid-gestation, there was a substantial increase in SCGN+ cells in the proliferative VZ and SVZ. Previous studies have provided evidence that, at this stage of development, there is an increased incidence of neurogenesis of GABAergic neurons, particularly those expressing calretinin, within the cerebral cortex itself (Letinic et al., 2002; Petanjek et al., 2009; Jakovcevski et al., 2011; Hladnik et al., 2014) therefore it is possible that some of these SCGN+ cells could be proliferative, or derived from proliferating cells in the dorsal telencephalon. Even though there is still appreciable Ki67 expression in the VZ at mid-gestation (Petanjek and Kostović, 2012; Alzu’bi and Clowry, 2019) we would conclude that the proportion of Ki67+ cells that are GABAergic neuron progenitors must be quite small as at 19PCW nearly all SCGN+ cells express DLX2 (a marker for GABAergic neuron progenitors and neuroblasts, see Figure 3) but nearly all DLX2+ cells are KI67− (i.e., non-proliferative, Alzu’bi and Clowry, 2019).

In summary, we have observed that approximately 60% of all cortical GABAergic neuron precursors (defined by DLX2 expression) co-express SCGN by mid-gestation in human, compared to only 45% that express CalR (this study) and up to 50% that is estimated to derive from the human CGE (Hansen et al., 2013) in total. This suggests the SCGN+ positive interneurons form the most populous subgroup of interneurons in the human neocortex. However, this is potentially a very diverse population as SCGN neurons can derive from the POA, this may include some cells with an MGE-like phenotype, that is, expressing parvalbumin and somatostatin when mature. It appears that SCGN expression may confer the increased capacity for neurite outgrowth (Raju et al., 2018) or specific migratory abilities to a large but diverse population of GABAergic neurons.

### Expression of MMP2 and ANXA5

Although, we found evidence of SCGN being co-expressed with either MMP2 or ANXA5, it was more striking to note the extensive independent expression of these three proteins. Even though MMP2 is proposed to promote neuroblast migration and neurite outgrowth by remodeling the local extracellular matrix (ECM; Lee et al., 2006; Mao et al., 2016; Hanics et al., 2017) it showed strong localization to the CP where cortical neurons stop migrating and where the cells are densely packed with little neurite outgrowth, or ingrowth of afferents, at this stage of development (Bystron et al., 2008). Furthermore, the effect of MMP2 on neural progenitors appears to be to maintain proliferation and prevent differentiation (Sinno et al., 2013; Shu et al., 2018) therefore expression of MMP2 in the CP appears paradoxical. However, perhaps secretion of MMP2 is required for neurons destined to inhabit the upper layers of the CP to migrate past densely packed neurons already stationary in the lower layers.

ANXA5 principally showed strong expression in the endothelial cells of blood vessels in the cortical wall. ANXA5 is secreted from endothelial cells where it interacts with phosphatidylserine (PS) abnormally expressed on the exofacial part of cell surface membrane in a Ca^2+^ dependent way to regulate coagulation and thus inhibit thrombosis (Flaherty et al., 1990; Van Heerde et al., 1994; Rao and Pendurthi, 2012). Our observations suggest it may also play this role in the developing cerebral vasculature. However, ANXA5 was also expressed by neuroblasts and neuronal progenitors in the proliferative zones. ANXA5 can bind to PS on the surface of cells undergoing apoptosis, inhibiting this process (Van Genderen et al., 2008) and in the brain the presence of ANXA5 in the ECM is indicative of ongoing neuronal death (Hefter et al., 2016). Thus, ANXA5 may play a role in modulating cell death during cortical development. ANXA5 was expressed by more mature neurons but not exclusively by SCGN neurons or neurons that co-expressed MMP2. At the early stages of CP development, ANXA5 showed the strongest expression in both the proliferative zones and in the more mature MZ but intriguingly, not the developmentally similar presubplate.

By mid-gestation intense expression was observed in a small subset only of cells resembling migrating neurons in the CP but was more extensively expressed in cells in the upper subplate (SP) at the boundary with the CP. The SP is known to contain a rich ECM, and by mid-gestation can be divided into layers containing cells of different phenotypes (Wang et al., 2010; Kostović et al., 2019). The upper SP contains a higher cell density than other SP strata (Kostović et al., 2019) and a higher density of acetylcholinesterase expressing thalamic and basal forebrain afferents (Kostović and Goldman Rakić, 1983; Kostović and Rakić, 1984) and synapses made between afferents, SP neurons and dendrites descending from the lower levels of the CP (Mrzljak et al., 1988; Kostović and Rakić, 1990) although, by this stage, it does not appear to be expressed in the other synapse dense region, the MZ (Figure 5). We would tentatively suggest that in this situation, ANXA5 is functioning intracellularly in active neurons in calcium-dependent signaling.

We have found evidence for SCGN+ neurons that co-expressed either ANXA5 or MMP2 and these were predominantly found within the SVZ. It seems probable (although not proven here) that SCGN+ neurons express both these proteins and may behave like cells of the adult RMS, remodeling the local ECM in response to CA^2+^ signaling to permit migration of these cells and/or other cells through this region which at this stage of development makes the transition from a predominantly proliferative zone to predominantly a zone for tangential migration of late-born GABAergic neurons (Alzu’bi and Clowry, 2019).

## Conclusions

The expression of SCGN by a considerable proportion of human cortical GABArgic neuron precursors of potentially diverse mature phenotypes is a striking but enigmatic observation. Raju et al.’s (2018) demonstration that it may confer the capacity for more extensive dendritic tree formation than found in corresponding rodent interneurons is persuasive but does not address why SCGN is expressed from the earliest stages of post-mitotic development. SCGN may also play a role in interneuron migration and perhaps in the elaboration of migratory routes we have observed in humans compared to the rodent cortex (Alzu’bi et al., 2017; Alzu’bi and Clowry, 2019; Molnár et al., 2019).

## Data Availability Statement

The datasets presented in this study can be found in online repositories. The names of the repository/repositories and accession number(s) can be found below: https://www.ebi.ac.uk/arrayexpress/; www.ebi.ac.uk/arrayexpress/experiments/E-MTAB-4840.

## Ethics Statement

The studies involving human participants were reviewed and approved by the Newcastle and North Tyneside NHS Health Authority Joint Ethics Committee. The patients/participants provided their written informed consent to participate in this study.

## Author Contributions

GC contributed to study design, data analysis and manuscript preparation. AA contributed to study design, data collection, data analysis and manuscript preparation.

## Conflict of Interest

The authors declare that the research was conducted in the absence of any commercial or financial relationships that could be construed as a potential conflict of interest.

## Footnotes

^1^https://www.hdbr.org

^2^www.ebi.ac.uk/arrayexpress/experiments/E-MTAB-4840

## Abbreviations

COUPTF-II: chicken ovalbumin upstream promoter transcription factor 2
DLX2: distal-less homeobox 2
GAD 67: glutamate decarboxylase isoform molecular weight 67 kD
GABA: gamma-aminobutyric acid
LHX6: LIM/homeobox protein 6
NKX2.1: NK2 homeobox 1
RNAseq: sequencing of total RNA species isolated from tissues
RPKM: Reads Per Kilobase of the transcript, per Million, mapped reads
SNAP25: synaptosome associated protein 25 kD
SP8: specificity protein 8.
